# Supplementary material for: Dysbiosis of human gut microbiome in young-onset colorectal cancer
Source: Nat Commun. 2021 Nov 19;12:6757. doi: 10.1038/s41467-021-27112-y (PMC8604900; doi:10.1038/s41467-021-27112-y)
Supplement: Supplementary file 1 — Supplementary Information [file 41467_2021_27112_MOESM1_ESM.pdf]

Dysbiosis of human gut microbiome in young-onset colorectal cancer

Yongzhi Yang[1,2#], Lutao Du[3#], Debing Shi[1,2#], Cheng Kong[4#], Jianqiang Liu[5#], Guang Liu[6#], Xinxiang Li[1,2], and Yanlei Ma[1,2]*

[1] Department of Colorectal Surgery, Fudan University Shanghai Cancer Center, Shanghai, China

[2] Department of Oncology, Shanghai Medical College, Fudan University, Shanghai, China

[3] Department of Clinical Laboratory, The Second Hospital of Shandong University, Jinan, 250033, Shandong province, China.

[4] Department of GI Surgery, Shanghai Tenth People's Hospital Affiliated to Tongji University, Shanghai, China

[5] Department of Endoscopy, Fudan University Shanghai Cancer Center, Shanghai, China.

[6] Quantum Hi-Tech Microecological Medical (Guangdong) Co.,Ltd.

[#] These authors contributed equally

[*] Corresponding author: Yanlei Ma, MD and PhD, Department of Oncology, Shanghai Medical College of Fudan University; Department of Colorectal Surgery, Fudan University Shanghai Cancer Center, No.270 Dongan' Road, Xuhui District, Shanghai 200032, China; E-mail: yanleima@fudan.edu.cn.

Supplementary information

Supplementary Figure Legends

Supplementary Figure 1. Gut microbiome across tumor stages and among tumor location in yCRC and oCRC.

Supplementary Figure 2. Phylogenetic profiles of fecal microbial communities in yCRC and oCRC detected by both 16S rRNA gene sequencing and metagenomic sequencing.

Supplementary Figure 3. Fecal microbial functional dysbiosis in yCRC and oCRC.

Supplementary Table Legends

Supplementary Table 1. Clinical characteristics of the enrolled participants for 16S rRNA gene sequencing in Fudan cohort (n = 728).

Supplementary Table 2. Clinical characteristics of the enrolled participants for metagenomic sequencing in Fudan cohort (n = 200).

Supplementary Table 3. Clinical characteristics of the enrolled participants for 16S rRNA gene sequencing in Huadong cohort (n = 310).

Supplementary Figure 1

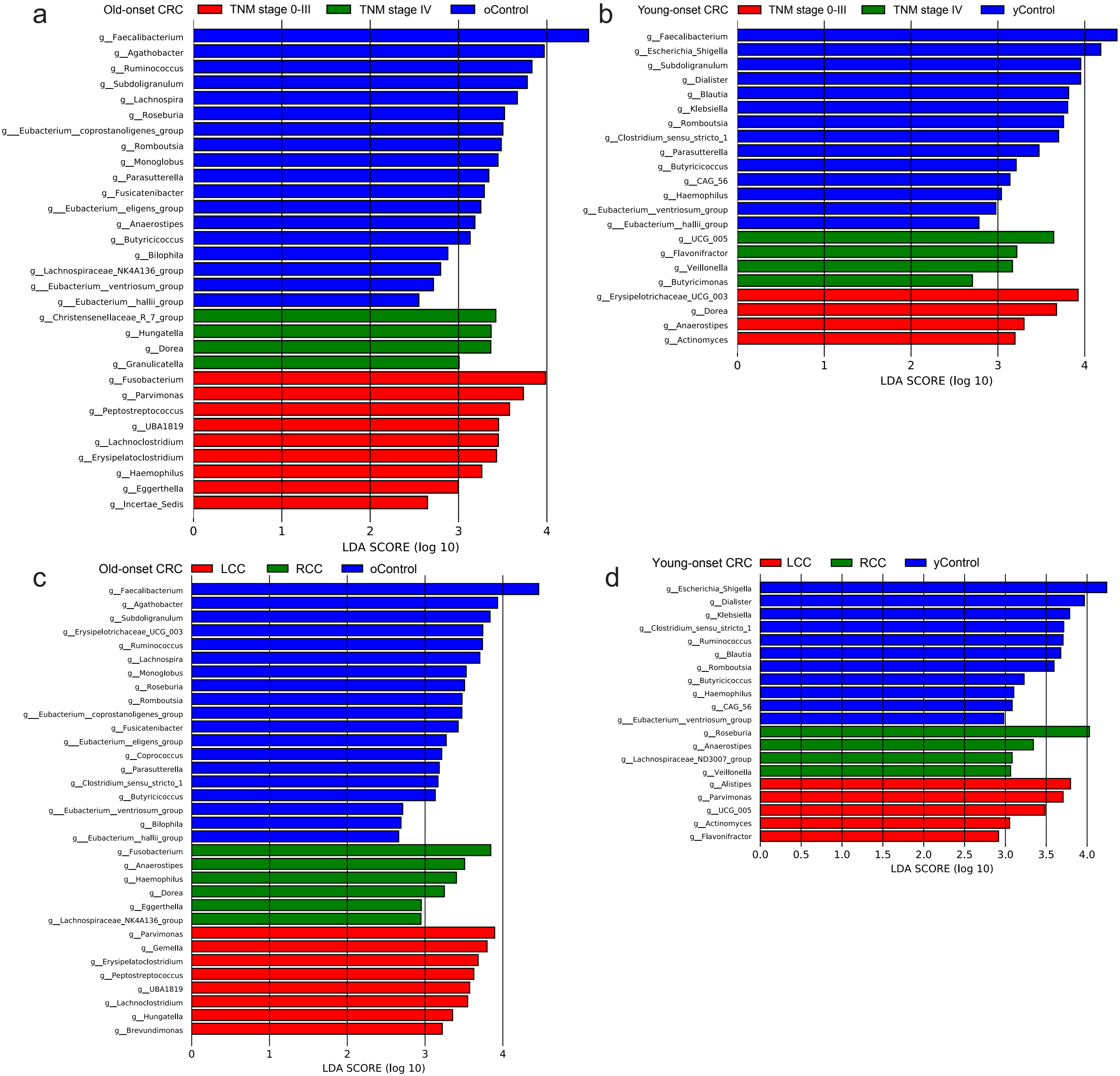

Supplementary Figure 1. **Gut microbiome across tumor stages and among tumor location in yCRC and oCRC.**

(a) Histogram of LEfSe measurement based on the 16S rRNA gene sequencing between 203 oControl and 233 old-onset CRC in different tumor stages. *p* values are calculated by Kruskall-Wallis test, LDA score >2.0, $p < 0.05$. **(b)** Histogram of LEfSe measurement based on the 16S rRNA gene sequencing between 148 yControl and 144 young-onset CRC in different tumor stages. *p* values are calculated by Kruskall-Wallis test, LDA score >2.0, $p < 0.05$. **(c)** Histogram of LEfSe measurement based on the 16S rRNA gene sequencing between 203 oControl and 233 old-onset CRC in different tumor locations. *p* values are calculated by Kruskall-Wallis test, LDA score >2.0, $p < 0.05$. **(d)** Histogram of LEfSe measurement based on the 16S rRNA gene sequencing between 148 young healthy control and 144 young-onset CRC in different tumor locations. *p* values are calculated by Kruskall-Wallis test, LDA score >2.0, $p < 0.05$. LDA, linear discriminant analysis; CRC, colorectal cancer; yCRC, young-onset CRC; oCRC, old-onset CRC; yControl, age-matched healthy controls for the yCRC; oControl, age-matched healthy controls for the oCRC; LCC, left-side colon cancer; RCC, right-side colon cancer. Source data are provided as a Source Data file.

Supplementary Figure 2

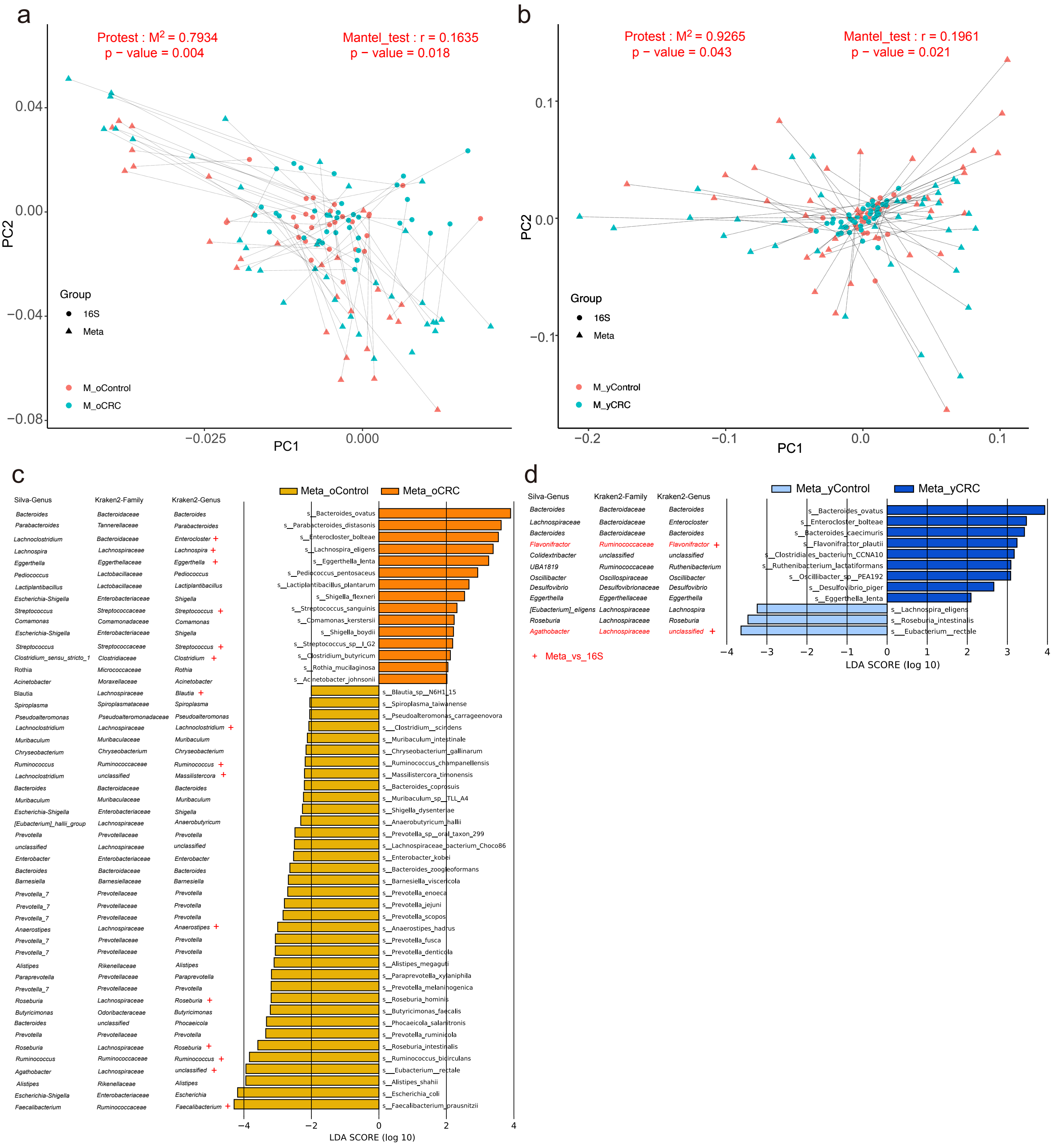

Supplementary Figure 2. Phylogenetic profiles of fecal microbial communities in yCRC and oCRC detected by both 16S rRNA gene sequencing and metagenomic sequencing.

(a-b) Procrustes plot comparing PCoA analysis results for 16S rRNA gene sequencing and metagenomic sequencing between oCRC and oControl (a), and between yCRC and yControl (b). Lines connect samples from the same subject. n = 50 per group. PROcrustean randomization test (Protest test) and Mantle test were performed to test for significance. M^2 value and r value indicated, two-sided. (**c-d)** Histogram of LDA coupled with effective size measurement in species level based on the metagenomic sequencing between oCRC and oControl (c), and between yCRC and yControl (d). n = 50 per group. *p* values are calculated by Kruskall-Wallis test, logarithmic LDA score > 2.0, $p < 0.05$. '+' showed bacterial genera with distinct relative abundances between groups detected by both 16S rRNA gene sequencing and metagenomic sequencing. LDA, linear discriminant analysis; CRC, colorectal cancer; yCRC, young-onset CRC; oCRC, old-onset CRC; yControl, age-matched healthy controls for the yCRC; oControl, age-matched healthy controls for the oCRC; PCoA, principal coordinate analysis. 16S, 16S rRNA gene sequencing data; Meta, metagenomic sequencing data. Source data are provided as a Source Data file.

Supplementary Figure 3

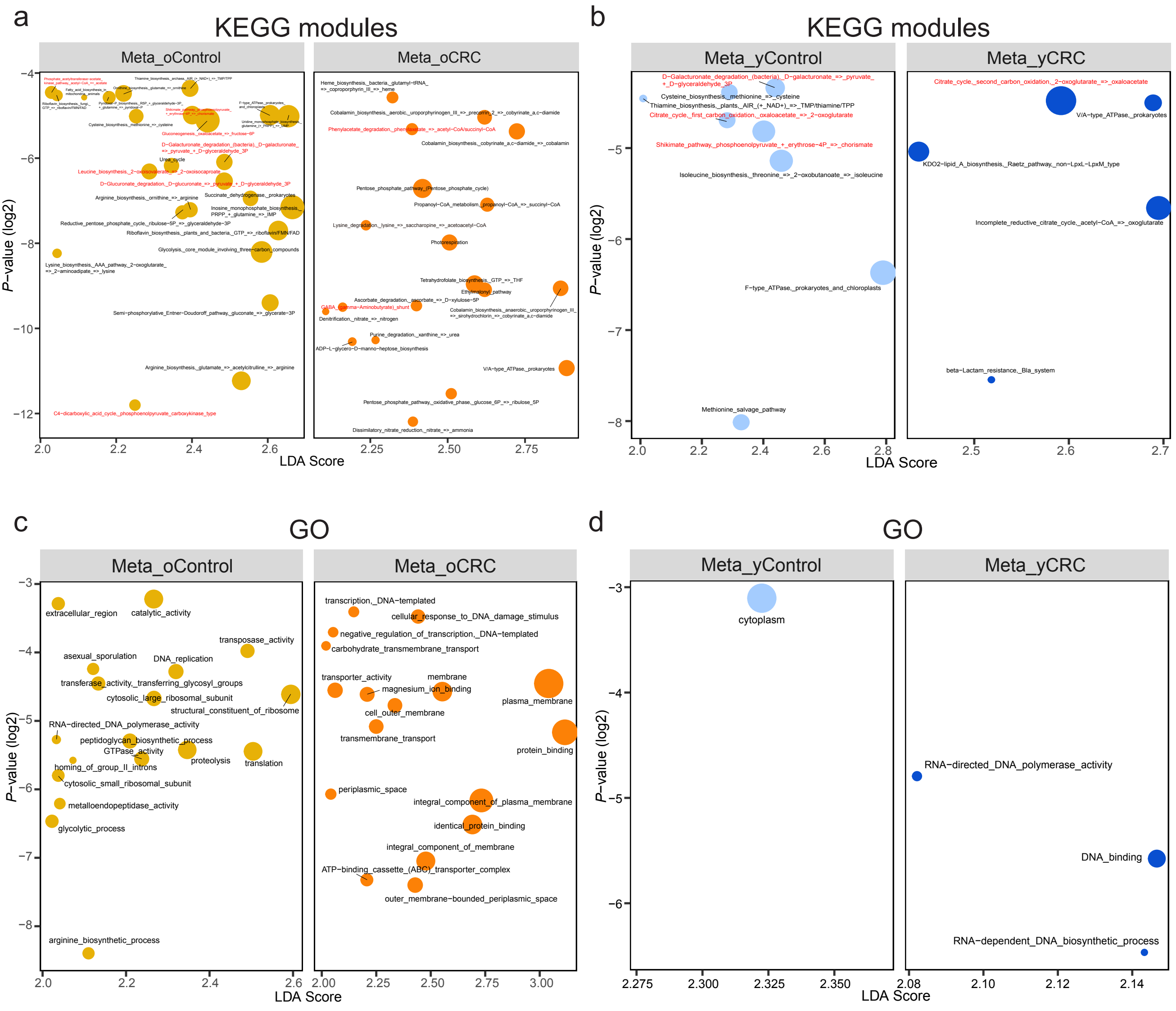

Supplementary Figure 3. **Fecal microbial functional dysbiosis in yCRC and oCRC.** **a-b** Fecal microbial functions between oControl and oCRC (a), and between yControl and yCRC (b) were predicted based on KEGG modules. n = 50 per group. *p* values are calculated by Kruskall-Wallis test, LDA score >2.0, $p < 0.05$. **c-d** Fecal microbial functions between oControl and oCRC (c), and between yControl and yCRC (d) were predicted based on GO. n = 50 per group. *p* values are calculated by Kruskall-Wallis test, LDA score >2.0, $p < 0.05$. LDA, linear discriminant analysis; CRC, colorectal cancer; yCRC, young-onset CRC; oCRC, old-onset CRC; yControl, age-matched healthy controls for the yCRC; oControl, age-matched healthy controls for the oCRC; KEGG, Kyoto Encyclopedia of Genes and Genomes; GO, Gene Ontology. Source data are provided as a Source Data file.

Supplementary Table 1. Clinical characteristics of the enrolled participants in Fudan cohort (n = 728)

Characteristics	oControl, n	oCRC, n	yControl, n	yCRC, n	*P* value (oControl vs oCRC)	*P* value (yControl vs yCRC)	*P* value (oCRC vs yCRC)
All participants (n)	203	233	148	144	N/A	N/A	N/A
Age					0.2137[a]	0.372[a]	< 0.0001[a]
Mean ± SD	63.23 ± 8.56	64.26 ± 8.68	39.76 ± 6.11	40.45 ± 7.02			
Median (Range, 25% - 75% percentile)	63 (41, 55 - 69)	64 (37, 57 - 70)	41 (25, 35 - 45)	43 (33, 36 - 46)			
Sex					0.0091	0.9068	0.1098
Female	109	96	72	72			
Male	94	137	76	72			
Tumor location					N/A	N/A	0.3758
Right hemicolon	N/A	45	N/A	30			
Left hemicolon	N/A	73	N/A	41			
Rectum	N/A	115	N/A	73			
Tumor size (cm)					N/A	N/A	0.8209
<5	N/A	157	N/A	99			
⩾5	N/A	76	N/A	45			
Differentiation					N/A	N/A	0.7251
Well-moderate	N/A	169	N/A	102			
Poor	N/A	64	N/A	42			
TNM stage					N/A	N/A	0.5319
0/I	N/A	40	N/A	21			
II	N/A	63	N/A	35			
III	N/A	57	N/A	45			
IV	N/A	73	N/A	43			
KRAS/NRAS/BRAF mutation					N/A	N/A	0.6595
Negative	N/A	166	N/A	98			
Positive	N/A	62	N/A	41			
NA	N/A	5	N/A	5			
Non-quantitative FOBT					< 0.0001	< 0.0001	0.8197
Negative	201	158	147	100			
Positive	2	75	1	44			
Serum CEA (mg/L)					< 0.0001	< 0.0001	0.1612
⩽5.9	201	131	147	92			
>5.9	2	102	1	52			
Serum CA19-9 (kU/L)					< 0.0001	< 0.0001	0.9043
⩽37	201	171	147	107			
>37	2	62	1	37			
Lymphatic invasion					N/A	N/A	0.1375
Negative	N/A	116	N/A	60			
Positive	N/A	117	N/A	84			
Nerve invasion					N/A	N/A	0.6529

Characteristics	oControl, n	oCRC, n	yControl, n	yCRC, n	*P* value (oControl vs oCRC)	*P* value (yControl vs yCRC)	*P* value (oCRC vs yCRC)
Negative	N/A	159	N/A	95			
Positive	N/A	74	N/A	49			
Vascular invasion					N/A	N/A	0.9999
Negative	N/A	133	N/A	83			
Positive	N/A	100	N/A	61			

Abbreviations: CRC, colorectal cancer; yCRC, young-onset CRC; oCRC, old-onset CRC; yControl, age-matched healthy controls for the yCRC; oControl, age-matched healthy controls for the oCRC; SD, standard deviation; FOBT, fecal occult blood test, N/A, not available. P values calculated by two-tailed unpaired Student's t test ([a]) or Pearson's Chi-square test. $p < 0.05$ considered statistically significant.

Supplementary Table 2. Clinical characteristics of the enrolled participants for metagenomic sequencing in Fudan cohort (n = 200)

Characteristics	oControl, n	oCRC, n	yControl, n	yCRC, n	*P* value (oControl vs oCRC)	*P* value (yControl vs yCRC)	*P* value (oCRC vs yCRC)
All participants (n)	50	50	50	50	N/A	N/A	N/A
Age					0.9031[a]	0.9379[a]	< 0.0001[a]
Mean ± SD	63.36 ± 9.67	63.58 ± 8.29	40.76 ± 6.09	40.66 ± 6.69			
Median (Range, 25% - 75% percentile)	61 (38, 55 - 70)	62 (32, 58 - 69)	41.5 (24, 37 - 45)	42.5 (24, 36 - 46)			
Sex					0.0228	0.5487	0.0601
Female	25	13	27	23			
Male	25	37	23	27			
Tumor location					N/A	N/A	0.4365
Right hemicolon	N/A	7	N/A	12			
Left hemicolon	N/A	15	N/A	14			
Rectum	N/A	28	N/A	24			
Tumor size (cm)					N/A	N/A	0.3342
<5	N/A	37	N/A	41			
⩾5	N/A	13	N/A	9			
Differentiation					N/A	N/A	0.8174
Well-moderate	N/A	37	N/A	38			
Poor	N/A	13	N/A	12			
TNM stage					N/A	N/A	0.1239
0/I	N/A	10	N/A	4			
II	N/A	19	N/A	14			
III	N/A	10	N/A	17			
IV	N/A	11	N/A	15			
KRAS/NRAS/BRAF mutation					N/A	N/A	0.6769
Negative	N/A	33	N/A	31			
Positive	N/A	17	N/A	19			
NA	N/A	0	N/A	0			
Non-quantitative FOBT					< 0.0001	< 0.0001	0.9999
Negative	50	29	49	29			
Positive	0	21	1	21			
Serum CEA (mg/L)					< 0.0001	< 0.0001	0.2797
⩽5.9	49	32	50	37			
>5.9	1	18	0	13			

Characteristics	oControl, n	oCRC, n	yControl, n	yCRC, n	***P* value (oControl vs oCRC)**	***P* value (yControl vs yCRC)**	***P* value (oCRC vs yCRC)**
Serum CA19-9 (kU/L)					< 0.0001	< 0.0001	0.2978
≤37	50	43	49	39			
>37	0	7	1	11			
Lymphatic invasion					N/A	N/A	0.1096
Negative	N/A	29	N/A	21			
Positive	N/A	21	N/A	29			
Nerve invasion					N/A	N/A	0.5235
Negative	N/A	35	N/A	32			
Positive	N/A	15	N/A	18			
Vascular invasion					N/A	N/A	0.3093
Negative	N/A	32	N/A	27			
Positive	N/A	18	N/A	23			

Abbreviations: CRC, colorectal cancer; yCRC, young-onset CRC; oCRC, old-onset CRC; yControl, age-matched healthy controls for the yCRC; oControl, age-matched healthy controls for the oCRC; SD, standard deviation; FOBT, fecal occult blood test, N/A, not available. P values calculated by two-tailed unpaired Student's t test (a) or Pearson's Chi-square test. $p < 0.05$ considered statistically significant.

Supplementary Table 3. Clinical characteristics of the enrolled participants in Huadong cohort (n = 310)

Characteristics	oControl, n	oCRC, n	yControl, n	yCRC, n	***P*** **value (oControl vs oCRC)**	***P*** **value (yControl vs yCRC)**	***P*** **value (oCRC vs yCRC)**
All participants (n)	54	146	69	41	N/A	N/A	N/A
Age					0.1024[a]	0.135[a]	< 0.0001[a]
Mean ± SD	60.46 ± 6.94	62.42 ± 7.67	37.74 ± 6.19	39.68 ± 7.11			
Median (Range, 25% - 75% percentile)	60 (26, 54-65)	62 (32, 55 - 68)	37 (33 - 42)	43 (25, 33 - 45)			
Sex					0.2644	0.114	0.5911
Female	28	62	37	15			
Male	26	84	32	26			
Tumor location					N/A	N/A	0.3059
Right hemicolon	N/A	45	N/A	10			
Left hemicolon	N/A	39	N/A	16			
Rectum	N/A	62	N/A	15			
TNM stage					N/A	N/A	0.1867
0/I	N/A	33	N/A	10			
II	N/A	50	N/A	7			
III	N/A	57	N/A	22			
IV	N/A	6	N/A	2			
Non-quantitative FOBT					< 0.0001	< 0.0001	0.2811
Negative	54	85	69	28			
Positive	0	61	0	13			
Serum CEA (ng/mL)					< 0.0001	< 0.0001	0.4599
≤5.9	53	92	68	29			
>5.9	1	54	1	12			
Serum CA19-9 (kU/L)					< 0.0001	< 0.0001	0.3601
≤37	54	117	69	36			
>37	0	29	0	5			

Abbreviations: CRC, colorectal cancer; yCRC, young-onset CRC; oCRC, old-onset CRC; yControl, age-matched healthy controls for the yCRC; oControl, age-matched healthy controls for the oCRC; SEM, standard error of mean; FOBT, fecal occult blood test; N/A, not available. P values calculated by two-tailed unpaired Student's t test ([a]) or Pearson's Chi-square test. $p < 0.05$ considered statistically significant.
