# Supplementary material for: Dysbiosis of human gut microbiome in young-onset colorectal cancer
Source: Nat Commun. 2021 Nov 19;12:6757. doi: 10.1038/s41467-021-27112-y (PMC8604900; doi:10.1038/s41467-021-27112-y)
Supplement: Supplementary file 2 — Description of Additional Supplementary Files [file 41467_2021_27112_MOESM2_ESM.pdf]

Description of Supplementary Data files

File Name: Supplementary Data 1

Description: The annotation of amplicon sequence variants in Fudan cohort.

File Name: Supplementary Data 2

Description: The annotation of amplicon sequence variants in Huadong cohort.

File Name: Supplementary Data 3

Description: The accession codes for the 16S rRNA gene sequencing data and the metagenomic sequencing data.
